# Preoperative oral dextromethorphan does not reduce pain or morphine consumption after open cholecystectomy

**DOI:** 10.4103/1658-354X.57876

**Published:** 2009

**Authors:** Hossein Mahmoodzadeh, Ali Movafegh, Noshin Mosavi Beigi

**Affiliations:** *Department of Surgery, Dr. Ali Shariati Hospital, Tehran University of Medical Sciences, Tehran, Iran*; 1 *Department of Anesthesiology and Critical Care, Dr. Ali Shariati Hospital, Tehran University of Medical Sciences, Tehran, Iran*; 2 *Department of Resident of Surgery, Dr. Ali Shariati Hospital, Tehran University of Medical Sciences, Tehran, Iran*

**Keywords:** *Dextromethorphan*, *postoperative pain*, *preemptive analgesia*

## Abstract

**Background:**

Dextromethorphan, the D-isomer of the codeine analog levorphanol, is a weak, noncompetitive N-Methyl-D-Aspartate (NMDA) receptor antagonist. It has been suggested that NMDA receptor antagonists induce preemptive analgesia when administered before tissue injury occurs, thus decreasing the subsequent sensation of pain.

**Materials and Methods:**

The study was conducted in the Dr. Ali Shariati Hospital, Tehran University of Medical Sciences, Tehran, Iran, between February 2005 and December 2006. In this study, 72 patients scheduled for elective cholesyctectomy were randomized into three groups to receive either oral dextromethorphan 45 mg (Group D45 = 24), dextromethorphan 90 mg (Group D90 = 24), or placebo (Group C, n = 24), as premedication, 120 minutes before surgery. A visual analog scale (VAS) for pain of each patient was measured at arrival in the ward and six and 24 hours after surgery.

**Results:**

The demographic characteristics of patients, ASA physical status class, duration of surgery, and the basal VAS pain score were similar in the two groups. There was no significant difference in the mean of the VAS pain scores measured over time or morphine consumption among the three groups.

**Conclusion:**

Dextromethorphan 45 mg and 90 mg, administrated orally, two hours before surgery, had no effect on postoperative morphine requirement and pain intensity.

## INTRODUCTION

Opioids are frequently administered to patients undergoing major surgery to alleviate postoperative pain, however, they may cause adverse effects such as nausea and vomiting, pruritus, urinary retention, and respiratory depression.[1]

As the analgesia and the side effects of opioids are dose-dependent a multimodal offset may enhance analgesia, while minimizing the side effects.[1]

Dextromethorphan (DM), the D-isomer of the codeine analog levorphanol, is a weak, noncompetitive N-Methyl-D-Aspartate (NMDA) receptor antagonist that has been used as an antitussive drug for more than 40 years.[2,3]

It has been suggested that NMDA receptor antagonists induce preemptive analgesia when administrated before tissue injury occurs, thus decreasing the subsequent sensation of pain.[2,3] There is good evidence from basic scientific literature to believe that acute post-injury pain in humans could be treated beneficially with a combination of opioids and NMDA antagonists.[4–6]

The purpose of the current study is to determine whether preoperative administration of 45 mg and 90 mg of oral DM would reduce postoperative pain and opioid consumption in comparison with a control group.

## MATERIALS AND METHODS

The study was conducted in the Dr. Ali Shariati Hospital, Tehran University of Medical Sciences, Tehran, Iran, between February 2005 and December 2006. The proposal was approved by the Institutional Ethics Committee and informed written consent was obtained from the patients. Seventy-two patients, 25-50 years, classified as ASA physical status I and II, who were undergoing open cholesyctectomy (midline approach) under general anesthesia, were enrolled in this randomized, double-blinded, and placebo-controlled study.

Patients who received opioids within 48 hours of surgery and sedatives or centrally acting drugs (central nervous system depressants or antidepressants) 21 days prior to surgery; those with a history of chronic pain, psychotic disorders or addiction including opioids, those with any contraindications to DM, and pregnant or lactating women were excluded from the study. All participants were given full explanations of DM and the visual analog scale for pain (VAS) on the day before surgery.

All drugs were given by an anesthesiologist who was not involved in patient observation, thus both the observer and patients were blinded to the group assignment. Patients were randomly assigned into three groups of either control (Group C, n = 24), DM 45 mg (Group D45, n = 24), or DM 90 mg, (Group D90, n = 24) using a computer-generated randomization list.

The patients in group C received placebo, group D45 received 45 mg DM, and those in group D90 received 90 mg DM orally, 120 minutes before surgery. Placebos were similar capsules containing sucrose.

On arrival in the operating room, all patients were monitored with an electrocardiogram (ECG), noninvasive blood pressure, and pulse oximetry. An 18-gauge cannula was inserted and lactated ringer solution 7 ml/kg was administered. Anesthesia was induced with 2 mg/kg propofol and 0.3 μg/kg sufentanyl; and endotracheal intubation was facilitated with 0.15 mg/kg cisatracurium. After tracheal intubation, anesthesia was maintained by isofluran and N_2_O (50%); 0.05 mg/kg cisatracurium and 0.2 μg/kg sufentanyl were administered half hourly. Ventilation was adjusted to maintain normocapnia (end-tidal carbon dioxide partial pressure 4.7-5.3 kPa). Patients were actively warmed to keep core temperature (esophageal) normothermic. At the start of the skin suturing, drug administration was stopped and the neuromuscular block was antagonized by IV administration of 2.5 mg of neostigmine, along with 1.25 mg of atropine. The patients were considered to be awake when they opened their eyes on demand or after gentle tactile stimulation; they were later extubated.

The severity of postoperative pain was measured and recorded using a 10 cm VAS, where 0 = no pain and 10 = the worst possible pain. The patients were asked to score the pain during coughing or movement at arrival in the ward, and 6 and 24 hours after surgery. The patients could request rescue analgesia at any time. Morphine, 0.1 mg/kg, was given as rescue analgesia at eight-hour intervals.

According to the previous studies, a sample size of 24 in each group would be sufficient to detect a difference of three scores in the mean of the pain score, and to estimate a power of 80% and a significance level of 5%. Statistical analysis was performed using SPSS package (SPSS Inc., Chicago, IL, USA), version 11.5. Normality of distribution was checked as needed. For statistical analysis of demographic data and for comparison of groups, one way ANOVA, repeated measure analysis of variance, Fishers exact or Chi-square tests were appropriate. Two tailed *P* < 0.05 was taken as significant.

## RESULTS

We randomized 72 patients. Three patients were excluded from the study because of surgical complications (two from C group and one from D45 group).

Demographic characteristics of patients, ASA physical status class, and the duration of surgery were similar in the two groups [Table 1].

**Table 1:** Patient characteristics

	Group control (n = 22)	Group DM 45 (n = 23)	Group DM 90 (n = 24)
Age (yr)*	48.3 ± 14.5	48.2 ± 14.3	46.2 ± 23.3
Sex (F/M)	13/9	13/10	14/10
Surgery time (minutes)*	98 ± 25.6	104 ± 23.2	112 ± 13.2
ASA class (I/II)	10/12	12/11	11/13
Weight (kg)*	78.3 ± 7.5	80.1 ± 10.4	75.1 ± 8.3

* Values are expressed as mean ± SD;

* There are no significant differences among the groups, DM - Dextromethorphan

There were no significant differences in the mean of the morphine consumption (12.39 ± 5.24 mg in the D45 group and 13.71 ± 4.28 mg in the D90 group versus 11.88 ± 5.29 in the C group) and the time for first morphine request (3.1 ± 1.7 hours in the D45 group and 3.2 ± 1.9 in the D90 group versus 2.9 ± 1.1 in the control group) [Table 2].

**Table 2:** VAS for pain, morphine consumption, and time for first morphine injection in groups

	Group control	Group DM45	Group DM90
Time for first morphine injection (hours)*	2.9 ± 1.1	3.1 ± 1.7	3.2 ± 1.9
Total morphine consumption (mg)*	11.88 ± 5.29	12.39 ± 5.24	13.71 ± 4.28
Visual analog scale for pain*	5.55 ± 1.38	4.73 ± 2.14	5.28 ± 2.81
Arriving ward			
6 hours	4.83 ± 2.39	4.22 ± 2.36	4.12 ± 2.80
24 hours	3.62 ± 2.92	4.00 ± 2.50	3.24 ± 2.95

* Values are expressed as mean ± SD;

* There are no significant differences among the groups, DM - Dextromethorphan, VAS: Visual analog scale

There was no significant difference in the mean of VAS pain scores measured over time among the three groups (5.55±1.38, 4.83±2.39, 3.62±2.92 in group C; 4.73 1 2.14, 4.22 1 2.36, 4.00 1 2.50 in group D45; 5.18±2.81, 4.12±2.80, 3.24±2.95 in group D90, repeated-measure analysis of variance, among-subjects, effects) [Table 2].

## DISCUSSION

The current study demonstrates that in patients undergoing open cholecystectomy, oral premedication with 45 or 90 mg DM or morphine consumption did not decrease postoperative pain intensity when compared with patients who received placebo.

Our findings are not consistent with the earlier reports that dextromethorphan reduces pain and analgesic requirement after various surgeries.[7–10]

N-methyl-D-aspartate receptor antagonism inhibits wind up or central hypersensitivity of the dorsal horn neurons in response to noxious stimulation.[2,11] Dextromethorphan, an NMDA receptor antagonist has been seen to reduce secondary hyperalgesia, but has no effect on primary hyperalgesia on healthy adult male volunteers.[2,12,13] Other investigators have been unable to demonstrate that DM, in a clinically relevant dose, has any effect on primary or secondary hyperanalgesia.[14,15]

The ability of DM to attenuate pain is controversial. Not all investigators agree that DM reduces opioid consumption or acute pain. Although DM has been used successfully as premedication for postoperative pain and morphine consumption reduction in some investigations,[6–10] other studies have not corroborated these reports.[16–19]

Ilkjaer *et al*. studied 50 patients undergoing non-malignant elective abdominal hysterectomy. The study was a double-blinded, randomized design that compared postoperative analgesia requirements and pain scores in patients who received preoperative DM or placebo. DM reduced morphine requirements in this sample and a modest (but non-significant) reduction in pain scores was found. They found that oral dextromethorphan 150 mg reduced PCA morphine consumption immediately (0-4 hours) after hysterectomy, without prolonged effects of pain or wound hyperalgesia.[18]

Thematic of the controversy surrounding the role of DM in acute pain management, Wadhaw *et al*. failed to demonstrate the specific opioid sparing effect expected from DM administration. In this 66 patient experimental sample, the investigators concluded that DM did not improve acute pain scores even at high doses.[19]

Many surgical procedures were included in both positive and negative studies, and there did not appear to be one specific procedure that yielded more benefit than the other. In the dextromethorphan studies, four negative studies used the oral route, and in two of these trials, at smaller doses of the drug, there was no direct analgesic effect of the intervention.[16–19]

The NMDA blocking properties of DM are probably less potent compared to ketamine.[2] It is possible that only subset of individuals will benefit from the NMDA properties of DM. It may also be that DM should be administered parenterally in a dose of at least 1 mg/kg for maximal preventive effect.[2]

Further investigations are required to determine whether larger doses or repeated doses of oral dextromethorphan will attenuate postoperative pain. However, undesirable side effects of dextromethorphan, including sedation and ataxia, are common in adults when the dose is increased above that recommended for antitussive therapy and may limit the usefulness of this strategy.[17]

The power analysis for this study indicated that there were sufficient numbers of patients in each group to detect 25% reduction in morphine use and 30 mm reduction in VAS for pain on moving.

## CONCLUSION

Dextromethorphan, 45 mg and 90 mg, administrated orally two hours before surgery had no effect on postoperative morphine requirement or pain intensity.
